# Emotional signals from faces, bodies and scenes influence observers' face expressions, fixations and pupil-size

**DOI:** 10.3389/fnhum.2013.00810

**Published:** 2013-12-18

**Authors:** Mariska E. Kret, Karin Roelofs, Jeroen J. Stekelenburg, Beatrice de Gelder

**Affiliations:** ^1^Psychology Department, University of AmsterdamAmsterdam, Netherlands; ^2^Cognitive Science Center Amsterdam, University of AmsterdamAmsterdam, Netherlands; ^3^Behavioural Science Institute & Donders Institute for Brain Cognition and Behaviour, Radboud University NijmegenNijmegen, Netherlands; ^4^Psychology Department, Cognitive and Affective Neurosciences Laboratory, Tilburg UniversityTilburg, Netherlands; ^5^Faculty of Psychology and Neuroscience, Cognitive Neuroscience, Maastricht UniversityMaastricht, Netherlands

**Keywords:** face expressions, body expressions, emotion, context, pupil dilation, fixations, electromyography

## Abstract

We receive emotional signals from different sources, including the face, the whole body, and the natural scene. Previous research has shown the importance of context provided by the whole body and the scene on the recognition of facial expressions. This study measured physiological responses to face-body-scene combinations. Participants freely viewed emotionally congruent and incongruent face-body and body-scene pairs whilst eye fixations, pupil-size, and electromyography (EMG) responses were recorded. Participants attended more to angry and fearful vs. happy or neutral cues, independent of the source and relatively independent from whether the face body and body scene combinations were emotionally congruent or not. Moreover, angry faces combined with angry bodies and angry bodies viewed in aggressive social scenes elicited greatest pupil dilation. Participants' face expressions matched the valence of the stimuli but when face-body compounds were shown, the observed facial expression influenced EMG responses more than the posture. Together, our results show that the perception of emotional signals from faces, bodies and scenes depends on the natural context, but when threatening cues are presented, these threats attract attention, induce arousal, and evoke congruent facial reactions.

## Introduction

Imagine a man approaches you while holding up his fists, his muscles tensed. Such an emotional signal is experienced differently in the context of a sports event than in a narrow street in the middle of the night. However, in the situation sketched above, one would most probably immediately *react*, and not actively stick a label on the man's emotional expression. The recognition of face expressions has received abundant attention in the emotion literature (Haxby et al., 2000; Adolphs, 2002). More recent studies have shown that our recognition of a facial expression is influenced by the body expression (Meeren et al., 2005; Van den Stock et al., 2007; Kret and de Gelder, 2013; Kret et al., 2013) and by the surrounding scene i.e., context (Righart and de Gelder, 2006, 2008a,b; Kret and de Gelder, 2012a). The goal of the current study is to examine how the presence of multiple emotional signals consisting of a simultaneously presented face and body expression, or a body expression situated in an emotional scene, are perceived by investigating the physiological correlates in a naturalistic passive-viewing situation.

When we observe another individual being emotional, different processes are initiated. First, our attention is drawn toward the face (Green et al., 2003; Lundqvist and Ohman, 2005; Fox and Damjanovic, 2006) and the body (Bandettini et al., 1992) as they contain the most salient information and usually complement each other. Next, we become aroused too: our heart beat changes, we start sweating, and our pupils dilate (Bradley et al., 2008). Moreover, it is likely that the observed emotion is reflected in our own face (Dimberg, 1982; Hess and Fischer, 2013). Thus, far, these physiological studies have mostly looked at the perception of isolated face expressions of emotion and not at all at the influence of a context such as the body posture. Investigating the perception of mixed messages from these different angles will contribute to the modification of existing models that attempt to predict the perception of incongruent emotion-context cues- but have failed so far (Mondloch et al., 2013). The present study aims to investigate two questions:

1. How are face and body expressions processed when presented simultaneously? Is a face looked at differently, depending on the body expression and vice-versa? Will the face expression and the level of arousal of the participant change as a function of the various emotional signals he observes in the face and body?
2. How are body expressions processed when presented in a social emotional context? Will the central figure be looked at differently, depending on the emotion of the social scene and vice-versa? Will the face expression and the level of arousal of the participant be different depending on the emotional signals from the body and scene?

In Experiments 1 and 2 we investigated the effects of context on physiological responses to face and body signals. Experiment 1 used realistic face-body compounds expressing emotionally congruent or incongruent signals of anger, fear, and happiness. We opted for these expressions for the following reasons. First, these three emotions can be expressed equally well via the body and the face contrary to surprise and disgust that are not well recognized from body expressions alone. Second, these emotions are all three arousing and contain a clear action component in the body expression (in contrast to a sad body expression). Third, anger, fear and happy expressions are the emotions that have been studied most often, and are also the ones we used in our previous studies in which we used similar experimental paradigms (yet with different dependent variables) (Kret and de Gelder, 2010, 2012a, 2013; Kret et al., 2011a,b,c, 2013). An angry expression can be interpreted as a sign of dominance. In contrast, fear may signal submissiveness. A smile can mean both. In the context of an aggressive posture, a smile is more likely to be interpreted as dominant, a laugh in the face. But when the body expresses fear, the smile may be perceived as an affiliative cue.

Experiment 2 used body-scene compounds, i.e., similar angry and happy body expressions, but combined with naturalistic social scenes showing emotionally congruent or incongruent angry, happy or neutral scenes. In Experiment 3 participants' recognition of body expressions was tested with the same stimuli as used in Experiment 2 to investigate whether body postures are better recognized in an emotionally congruent vs. incongruent context scene (Kret and de Gelder, 2010).

Regarding our first research question, we predicted that angry and fearful expressions, whether from the face or from the body would attract most attention which would be in line with previous studies that showed that angry cues grab the attention more than happy cues (Öhman et al., 2001; Green et al., 2003; Bannerman et al., 2009). Therefore, we expected longest fixation durations on angry bodies, especially when the simultaneously presented face showed a happy expression. Furthermore, we predicted that angry faces combined with angry bodies would elicit most pupil dilation values, as the presence of both cues may increase the overall perceived intensity of the stimulus. We expect this to be reflected in the face of the participant as well, i.e., greatest corrugator activity in response to angry faces combined with angry bodies, greatest zygomaticus activity when happy faces were combined with happy body expressions. Secondly, we hypothesized that gaze would be attracted by anger in the body and the scene and that attention would predominantly be allocated to an angry body presented in a neutral context, as a neutral context would pull least attention away from the body. In addition, we expected the greatest pupil dilation in response to stimuli that contain the most arousing cues, i.e., an angry body expression shown in an aggressive context and that the face of the participant would reflect the valence of the total scene including the foreground figure. In sum, we predict that participants' reactions are more influenced by emotional cues, and that multiple cues of the same emotion add up, than by incongruence between multiple cues.

## Results

### Experiment 1. face-body composite images

**Figure 1:**
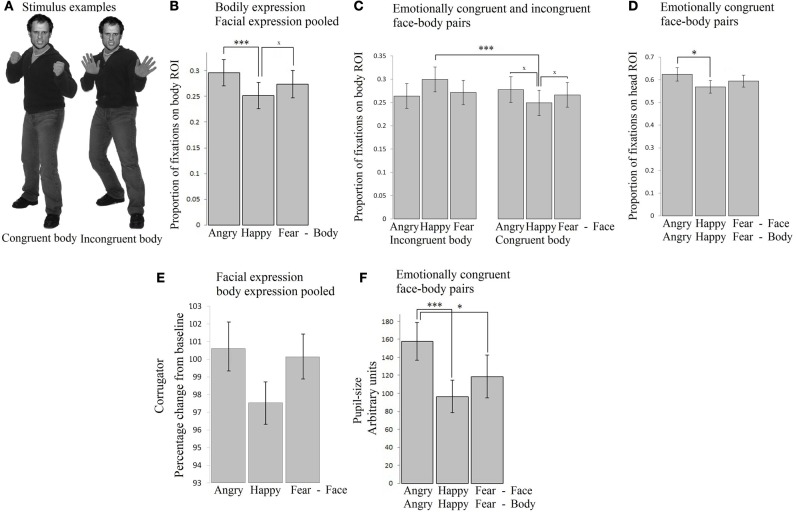
**Experiment 1**. Passive viewing of face - bodiy pairs **(A)** Stimulus examples **(B)** Fixation duration on bodies (body ROI) was mainly influenced by the body expression. Angry expressions induced longest fixations. **(C)** Fixation duration on bodies (body ROI) below happy faces were longer when the bodies expressed fear or anger, than when they expressed happiness. **(D)** Fixation duration on faces (face ROI) with congruent body expressions showed that angry faces were longer looked at than happy faces. **(E)** Corrugator responded to angry and fearful faces, independent of the body posture **(F)** Pupil-size was largest when observing anger simultaneously from the face and from the body. The error bars represent the mean standard error. ^×^*p* < 0.1; ^*^p < 0.05; ^**^p < 0.01; ^***^p < 0.005.

Participants freely viewed angry, happy, and fearful face expressions paired with body expressions in all combinations (angry face with angry, happy, and fearful body, happy face with angry, happy, and fearful body, fearful face with angry, happy, and fearful body). See Figure 1A for two stimulus examples. This experiment was set up to provide insight into how emotional signals from the body (body region of interest, ROI) and face (face ROI) are processed spontaneously and to what extent the expressions of the face and the body attract attention, induce arousal and face expressions in the observer. All the means and standard errors for all measures can be found in Supplementary Table 1.

#### Fixations on the body

A 3 × 3 (face expression × body expression) Repeated Measures ANOVA showed that within the body region of interest (ROI), we observed a main effect for body expression: fearful and angry bodies were looked at longer than happy bodies *F*_(2, 72)_ = 12.026, *p* < 0.001, η*p*^2^ = 0.250 [anger (*M* = 0.30, *SE* = 0.03) vs. happy (*M* = 0.25, *SE* = 0.03) *p* < 0.001; fear (*M* = 0.27, *SE* = 0.03) vs. happy *p* = 0.06]. There were no other main or interaction effects (see Figure 1B).

In order to test for congruency effects, we ran a 2 × 3 Repeated Measures ANOVA with congruence (congruent or incongruent) of the body signal × face expression (anger, fear, happy), and face expression, which yielded a significant interaction *F*_(2, 72)_ = 5.189, *p* < 0.01, η*p*^2^ = 0.126. A follow-up *t*-test revealed that bodies were longer looked at when they were emotionally incongruent vs. congruent with a happy facial expression (i.e., pooled anger/fear vs. happy body posture) *t*_(36)_ = 3.799, *p* = 0.001. When including just the congruent stimuli, we did not find a statistically significant effect of emotion, although a trend was observed, with somewhat more fixations attributed to the body ROI in case of anger vs. fear or happy postures *F*_(2, 72)_ = 2.330, *p* = 0.10, η*p*^2^ = 0.061 (see Figure 1C)^1^.

#### Fixations on the face

A 3 × 3 (face expression × body expression) Repeated Measures ANOVA unexpectedly showed that fixations on the face were not modulated by facial expressions and only showed statistical trends *F*_(2, 72)_ = 2.779, *p* = 0.069, η*p*^2^ = 0.072. The interaction between facial and body expression also showed a statistical trend toward significance *F*_(4, 144)_ = 2.212, *p* = 0.071, η*p*^2^ = 0.058. Further tests did not reveal significant differences. There was no main effect of body expression.

In order to test for congruency effects, we ran a 2 × 3 Repeated Measures ANOVA with congruence of the body signal, and face expression, which yielded a significant interaction *F*_(2, 72)_ = 4.272, *p* < 0.05, η*p*^2^ = 0.106. A follow-up *t*-test revealed that angry faces were somewhat longer looked at when paired with angry, than with happy or fearful bodies *t*_(36)_ = 1.951, *p* = 0.059. When including just the congruent stimuli in the Repeated Measures ANOVA, we did observe an effect of facial expression *F*_(2, 72)_ = 5.664, *p* = 0.005, η*p*^2^ = 0.136. In the congruent condition, angry faces were longer looked at than happy faces (*p* < 0.05) (see Figure 1D).

***EMG-corrugator.*** A 3 × 3 (face × body expression) Repeated Measures ANOVA showed a main effect of facial expression *F*_(2, 56)_ = 11.394, *p* < 0.001, η*p*^2^ = 0.289, corrugator activity showed a selective increase following angry and fearful (*M* = 100.61, *SE* = 1.06 and *M* = 100.14, *SE* = 1.06) vs. happy faces (*M* = 97.54, *SE* = 1.05) (*p*-values < 0.005). The interaction between bodily and facial expression was not significant but showed a trend *F*_(4, 112)_ = 2.087, *p* = 0.087, η*p*^2^ = 0.069. Further tests did not reveal any significant differences. There was no main effect of body expression. We found no indication of congruency effects, as was tested with a 2 × 3 (congruence × face expression) Repeated Measures ANOVA with congruence of the body signal, and face expression as factors (see Table 1). See Figure 1E.

**Table 1:** **Electromyography**.

**Body**	**Face**	**Zygomaticus**		**Corrugator**	
		**Mean**	***SE***	**Mean**	***SE***
Anger	Anger	106.520	2.477	99.415	0.955
	Happy	106.336	2.402	98.672	1.223
	Fear	111.678	4.836	98.916	1.285
Happy	Anger	109.806	3.147	100.933	1.606
	Happy	109.630	2.653	97.770	0.877
	Fear	110.976	7.288	101.136	1.228
Fear	Anger	110.472	6.674	101.468	1.462
	Happy	113.608	6.371	96.173	1.494
	Fear	109.965	5.457	100.355	1.252

***EMG-zygomaticus.*** The 3 × 3 (face × body expression) Repeated Measures ANOVA showed that the zygomaticus was equally responsive to all stimuli, i.e., there were no significant effects of face or body expression. There were no other main or interaction effects. We found no indication of congruency effects (see Table 1).

To test whether a lack of a main effect for bodies on the EMG responses was due to the short fixations on bodies, we computed correlations between fixation duration and zygomaticus and corrugator activity. We found no evidence for such a relationship. Other studies showed clear EMG responses to unseen stimuli, suggesting that fixation patterns should not influence EMG responses (Tamietto et al., 2009).

***Pupillometry.*** We analyzed pupil-size in a 3 × 3 Repeated Measures ANOVA. The results showed no main or interaction effects. In order to test for congruency effects, we ran a 2 × 3 Repeated Measures ANOVA with congruence of the body signal, and face expression, which yielded a significant interaction *F*_(2, 72)_ = 3.653, *p* < 0.05, η*p*^2^ = 0.092. Angry faces evoked greater pupil dilation when paired with angry than with fearful or happy bodies *t*_(36)_ = 2.610, *p* < 0.05. In a Repeated Measures ANOVA with just the emotionally congruent stimuli, a strong effect of emotion was observed *F*_(2, 72)_ = 5.701, *p* < 0.005. Observing angry persons (*M* = 157.86, *SE* = 20.99) evoked greater pupil dilation than observing fearful (*M* = 118.48, *SE* = 23.77) (*p* < 0.05) or happy persons (*M* = 96.44, *SE* = 23.77) (*p* < 0.005). There was no difference between fear and happiness (see Figure 1F).

To test whether a lack of a main effect for bodies on the pupil response was due to the short fixations on bodies, we computed correlations between looking times and pupil-size. We found no evidence for such a relationship. We also explored correlations between fixations on the head and pupil-size and found one significant negative correlation between fixation durations on the head-ROI of happy faces above fearful bodies and pupil-size (*r* = −0.452, *p* = 0.005, uncorrected, *p* = 0.045, Bonferroni-corrected). This finding is consistent with our finding that pupil-sizes were smallest following happy vs. angry or fearful cues so the longer participants fixated on happy cues, the smaller their pupil-sizes should be. These exploratory analyses can be found in Supplementary Table 2.

### Experiment 2. body-scene composite images

In Experiment 1, participants observed face-body composite images and we showed that participants' gaze was attracted to threatening cues from the body, that participants' pupils dilated mostly in response to congruent angry cues and that the corrugator reacted to angry and fearful faces but not bodies. In Experiment 2, the same participants viewed a new set of naturalistic stimuli consisting of angry and happy body expressions situated in angry, happy, and neutral social scenes.

We often encounter somebody in a context that includes other people. Especially when seeing someone being emotional, the context, and the social context in particular may contribute to understanding the emotion of the observed. The goal of Experiment 2 was to investigate how body expressions are processed when presented in a social emotional context. A figure with a happy or angry body expression facing the participant was placed in the middle of a crowd that consisted of other emotional or neutral figures. The central figure was easy to distinguish from the crowd as it appeared always in the middle of the scene, facing the observer. Key questions were whether the central figure would be looked at differently, depending on the emotion of the social scene and whether the face expression and the level of arousal of the participant would be different depending on the emotional signals from the presented body and scene.

#### Fixations on the body

A 2 × 3 (body expression × emotional scene) Repeated Measures ANOVA revealed an interaction between body and scene emotion on fixation duration within the body ROI *F*_(2, 72)_ = 3.991, *p* < 0.05, η*p*^2^ = 0.100. In a neutral scene, angry bodies were looked at longer than happy bodies [*t*_(36)_ = 3.120, *p* < 0.05]. In contrast, in emotional scenes, these differences disappeared. There were no main effects. No effects were found when we tested for congruency with only the emotional conditions (i.e., via a 2 × 2 Repeated Measures ANOVA without the neutral condition).

**Figure 2:**
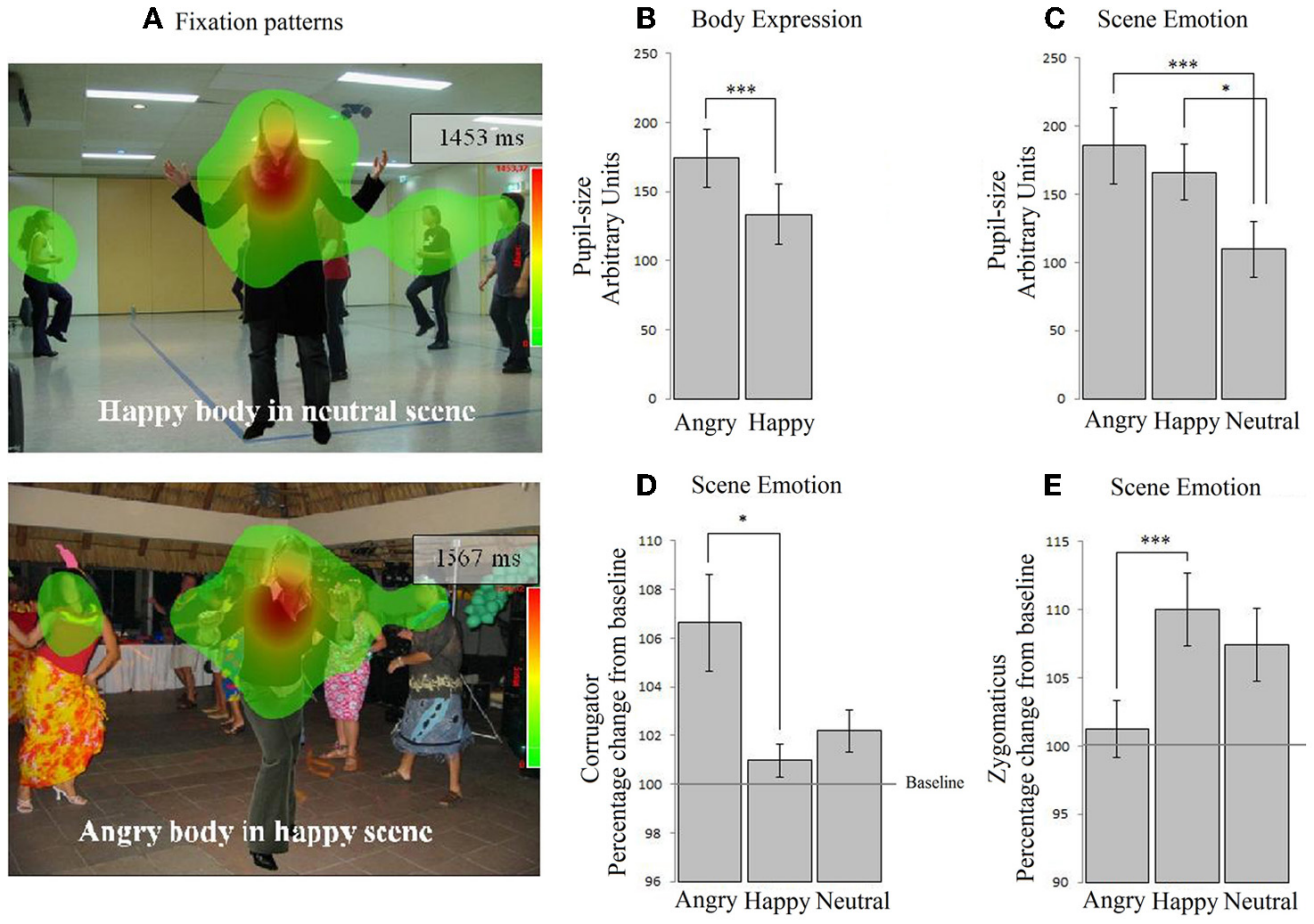
**Experiment 2**. Passive viewing of bodies in social scenes. **(A)** The two heat-maps show that participants fixated on the people in the scene. **(B)** Participants' pupils dilated mostly in response to angry cues, both from the body and from the scene **(C)**. **(D)** In contrast, the corrugator exclusively responded to angry scenes, not angry bodies. **(E)** Similarly, the zygomaticus responded to happy scenes and was unresponsive to body expressions. ^*^p < 0.05; ^***^p < 0.005.

We computed the duration of the fixations that fell on the hand region (*M* = 0.06, *SE* = 0.004). As most participants at least fixated on the hands once, we decided to further analyze this pattern. There were no main effects of bodily expression and no interaction between bodily expression and scene emotion on the fixations on the hand region. There was no significant effect of scene emotion, although a trend was observed *F*_(2, 72)_ = 2.360, *p* = 0.10, η*p*^2^ = 0.062 on the fixation duration on the hands, but follow-up comparisons did not show significant differences (*p* ≥ 0.08). No effects were found when we tested for congruency with only the emotional conditions (neutral scenes excluded) (see Table 2). See Figure 2A.

**Table 2:** **Fixation duration**.

**Body**	**Scene**	**Body**		**Scene**	**Hands**	
		**Mean**	***SE***		**Mean**	***SE***
Anger	Anger	0.408	0.027	Anger	0.062	0.004
	Happy	0.406	0.025	Happy	0.061	0.005
	Neutral	0.429	0.026	Neutral	0.054	0.005
Happy	Anger	0.410	0.024			
	Happy	0.399	0.027			
	Neutral	0.379	0.026			

***Pupil dilatation.*** A 2 × 3 (body × scene emotion) Repeated Measures ANOVA revealed main effects of body and of scene emotion *F*_(1, 36)_ = 8.873, *p* < 0.01, η*p*^2^ = 0.198; *F*_(2, 72)_ = 8.049, *p* < 0.005, η*p*^2^ = 0.183. Pupil-size was larger following angry vs. happy bodies (*M* = 174.08, *SE* = 21.11 vs. *M* = 133.56, *SE* = 21.63) (*p* < 0.005) and angry vs. neutral (*M* = 185.73, *SE* = 28.02 vs. 109.66, *SE* = 20.38) (*p* < 0.05) and happy vs. neutral (*M* = 166.07, *SE* = 20.46 vs. *M* = 109) (*p* < 0.001) scenes. There were no interactions. No effects were found when we tested for congruency with only the emotional conditions (see Figures 2B,C).

***EMG-corrugator.*** A 2 × 3 (body × scene emotion) Repeated Measures ANOVA revealed that the corrugator was more responsive to angry vs. happy scenes (*M* = 106.634, *SE* = 2.060 vs. 100.966, *SE* = 0.699) (*p* < 0.05) *F*_(2, 72)_ = 5.584, *p* < 0.01, η*p*^2^ = 0.134. There was no main effect of body expression and no interaction. No effects were found when we tested for congruency with only the emotional conditions (see Figure 2D).

***EMG-zygomaticus.*** A 2 × 3 (body × scene emotion) Repeated Measures ANOVA revealed that the zygomaticus showed an opposite response pattern *F*_(2, 72)_ = 7.858, *p* < 0.005, η*p*^2^ = 0.179 [more for happy (*M* = 110.004, *SE* = 2.653) vs. angry scenes (*M* = 101.257, *SE* = 2.093) (*p* < 0.005) and marginally significant for happy as compared to neutral (*M* = 107.413, *SE* = 2.653) *p* = 0.069]. There were no main effects or interactions with body expression. When we tested congruency by only including the emotional conditions, we found an interaction between body and scene congruence *F*_(1, 36)_ = 11.968, *p* = 0.001, η*p*^2^ = 0.250. The zygomaticus response was larger following happy bodies in happy than in angry scenes *t*_(36)_ = 2.378, *p* < 0.05 (see Figure 2E).

We explored possible relationships between fixation durations and EMG and pupil-responses, but did not find evidence for any relationship.

### Experiment 3. fast recognition of body expressions in body-scene composite images

After completion of Experiments 1 and 2, we showed the same participants the stimuli of Experiment 2 once more and asked them to categorize the body expression while ignoring the scene emotion, which was easy as the stimuli were only presented for 100 ms (Kret and de Gelder, 2010). We predicted to find a congruency effect in that participants could better recognize body expressions when presented in an emotionally congruent vs. emotionally incongruent context scene.

***Accuracy.*** A 2 × 3 (body × scene emotion) Repeated Measures ANOVA revealed that there was an interaction between body and scene emotion *F*_(2, 70)_ = 5.092, *p* < 0.01, η*p*^2^ = 0.127. Angry bodies were better recognized in an angry vs. happy context *t*_(35)_ = 2.477, *p* = 0.018 and happy bodies somewhat better in a happy vs. angry context, although this effect did not reach statistical significance *t*_(35)_ = 1.755, *p* = 0.088.

## Methods

### Participants Experiments 1–3

Thirty-seven participants (26 females, mean age 22.7, range 19–29 years old; 11 males; mean age: 23.8, range 19–32 years old) filled out an informed consent and took part in all three experiments and in additional emotion recognition tasks that are published elsewhere (Kret et al., 2013). Participants had no neurological or psychiatric history, were right-handed and had normal or corrected-to-normal vision. The study was performed in accordance with the Declaration of Helsinki and approved by the local medical ethical committee.

### Materials Experiment 1

Fearful, happy and angry face expressions of six male individuals that were correctly recognized above 80% were selected from the NimStim set (Tottenham et al., 2009). The corresponding body expressions were taken from our own stimulus database containing 254 digital pictures. The pictures were shot in a professional photo studio under constant lightening conditions. Non-professional actors were individually instructed in a standardized procedure to display four expressions (anger, fear, happiness, and sadness) with the whole body. The instructions provided a few specific and representative daily events typically associated with each emotion (for more details, see de Gelder and Van den Stock, 2011). For the current study, we selected the best actors, with recognition scores above 80% correct. We used only male faces and bodies because we previously found that these evoke stronger arousal when anger and fear are expressed (Kret et al., 2011a; Kret and de Gelder, 2012b). Pictures were presented in grayscale, against a gray background. Using Adobe Photoshop the luminance of each stimulus was adjusted to the mean. A final check was made with a light meter on the test computer screen. The size of the stimuli was 354 × 532 pixels. See Figure 1A for two examples.

### Procedure Experiment 1

After applying the electrodes on the participants face, the eye-tracking device was positioned on the participant's head. Next, a 9-point calibration was performed which was repeated before each block. Stimuli were presented using E-prime software on a PC screen with a resolution of 1024 by 768 and a refresh rate of 100 Hz. Each trial started with a fixation-cross, shown for minimally 3000 ms until the participant fixated and a manual drift correction was performed by the experiment leader, followed by a picture presented for 4000 ms and a gray screen (3000 ms). The stimuli were divided in two blocks containing 36 trials each with 18 congruent and 18 incongruent stimuli. To keep participants naive regarding the purpose of the electromyography (EMG), they were told that the electrodes recorded perspiration. Participants were asked to observe the pictures without giving a response. After the experiment, they were asked to describe what they had seen. All mentioned having seen emotional expressions but that sometimes the facial and body expressions were not the same.

Fixations, pupil dilation and EMG responses were analyzed in separate 3 × 3 (face expression × body expression) Repeated Measures ANOVAs. Fixations were analyzed per ROI (body, hands, and face ROI) that were defined by the pixels on the whole body (including the neck) and the pixels of the head. Significant main effects were followed up by Bonferroni-corrected pairwise comparisons, and interactions by Bonferroni-corrected 2-tailed *t*-tests.

### Materials Experiment 2

Stimulus materials consisted of congruent and incongruent body-scene pairs (see Figure 2A for examples). The pictures of bodies (from eight male actors, with the facial features blurred) were taken from the same set as those in Experiment 1 and expressed anger and happiness. The scenes (eight unique scenes per emotion condition) were selected from the Internet and showed angry, happy, or neutral scenes. The number of people in the different scenes was similar across emotion conditions. These scenes have been validated before in an emotion-recognition task and were recognized very accurately, even though they were presented only for 100 ms (anger 88%, happy 97%, and neutral 92%) (Kret and de Gelder, 2010). We here left out fearful bodies and scenes and included neutral scenes instead. Including anger, fear, happy, and neutral bodies and scenes would have yielded too many conditions.

We conducted an additional validation study among 36 students following standard validation procedures of Bradley and Lang (1999). Neutral scenes were rated as significantly calmer than happy scenes *t*_(35)_ = 4.098, *p* < 0.001 and as somewhat calmer than angry scenes *t*_(35)_ = 1.836, *p* = 0.075. Angry and happy scenes were equally emotionally intensive *t*_(35)_ = 0.462, *p* = 0.647 and were both more intensive as neutral scenes *t*_(35)_ = 4.298, *p* < 0.001; *t*_(35)_ = 7.109, *p* < 0.001.

The stimulus presentation duration and inter-trial interval of Experiment 2 were the same as in Experiment 1.

### Procedure Experiment 2

Half of the participants started with Experiment 1, and the other half with Experiment 2. The procedure of Experiment 2 was the same as for Experiment 1, except that there were 48 trials that were randomly presented within a single block. The data were analyzed in separate 2 (body emotions) × 3 (scene emotions) Repeated Measures ANOVAs.

### Procedure Experiment 3

After completion of Experiments 1 and 2, we showed the participants the stimuli of Experiment 2 once more, this time with a brief presentation duration (100 ms) and with the task to categorize the body expression while ignoring the scene emotion. The proportion correct responses was analyzed in a 2 (body emotions) × 3 (scene emotions) Repeated Measures ANOVA.

### Measurements

#### Facial EMG

The parameters for facial EMG acquisition and analysis were selected according to standard guidelines (Van Boxtel, 2010). BioSemi flat-type active electrodes were used and facial EMG was measured bipolarly over the zygomaticus major and the corrugator supercilii on the right side of the face at a sample rate of 1024 Hz. The common mode sense (CMS) active electrode and the driven right leg (DRL) passive electrode were attached to the left cheek and used as reference and ground electrodes, respectively (http://www.biosemi.com/faq.htm). Before attachment, the skin was cleaned with alcohol and the electrodes were filled with electrode paste. Raw data were first filtered offline with a 20–500 Hz band-pass in Brain Vision Analyzer Version 1.05 (Brain Products GmbH), and full-wave rectified. Data were visually inspected for excessive movement during baseline by two independent raters who were blind to the trial conditions. Trials that deemed problematic were discarded, resulting in the exclusion of 6.07% (*SD* 7.50) of the trials from subsequent analysis. Due to technical problems, the EMG data of four participants in Experiment 1 and three in Experiment 2 were not recorded. Subsequently, mean rectified EMG was calculated across a 4000-ms post-stimulus epoch, and a 1000 ms pre-stimulus baseline period. Mean rectified EMG was expressed as a percentage of the mean pre-stimulus baseline EMG amplitude. Percentage EMG amplitude scores were averaged across valid trials and across emotions.

The zygomaticus is predominantly involved in expressing happiness. The corrugator muscle can be used to measure the expression of negative emotions including anger and fear. But in order to differentiate between these two negative emotions, measuring additional face muscles such as the frontalis would be necessary (Ekman and Friesen, 1978). However, this was not possible in the current experiment, due to the head-mounted eye tracker. Activity of the corrugator in a specific context, such as by presenting clear emotional stimuli, can be interpreted as the expression of the observed emotion (Overbeek et al., 2012).

#### Eyetracking

Eye movements were recorded with a sample rate of 250 Hz using the head-mounted EyeLink Eye Tracking System (SensoMotoric Instruments GmbH, Germany). A drift correction was performed on every trial to ensure that data was adjusted for movement. We used the default Eyelink settings which defined a blink as a period of saccade-detector activity with the pupil data missing for three or more samples in a sequence. A saccade was defined as a period of time where the saccade detector was active for 2 or more samples in sequence and continued until the start of a period of saccade detector inactivity for 20 ms. The configurable acceleration (8000°/s) and velocity (30°/s) threshold were set to detect saccades of at least 0.5° of visual angle. A fixation was defined as any period that was not a blink or saccade. Analyses were performed on the proportion of time spent looking at each ROI within the time spent looking on the screen, with the first 200 ms discarded due to the fixed position of the fixation cross. In accordance with previous literature, a 500 ms baseline was subtracted from all subsequent data-points. Missing data due to blinks were interpolated linearly. The first 2 s of the pupillary response were not included in the analysis to avoid influences of the initial dip in pupil-size (Bradley et al., 2008; Kret et al., 2013).

## Discussion

We investigated the perception of emotional expressions using naturalistic stimuli consisting of whole body expressions and scenes. Two main findings emerge from the studies: (1) Observers' reactions to face and body expressions are influenced by whole body expressions and by the surrounding social scene. Thus the perception of face and body expressions is influenced by the natural viewing conditions of the face and body. (2) When people are confronted with threat, be it from the face, the body, or the scene, participants' pupils dilated, their corrugator muscle became more active and they directed their gaze to the threat. These conclusions are based on the results of two main experiments. In Experiment 1, emotionally congruent and incongruent face-body pairs were shown. Experiment 2 showed emotionally congruent and incongruent body-scene pairs. Critically and uniquely in both experiments we combined EMG, pupil responses as well as fixations on faces, bodies, and scenes, and in addition we tested subjective emotional ratings (Experiment 3). Our main findings support the motivated attention theory (Lang and Cuthbert, 1997; Bradley et al., 2003). In line with this theory, visual attention, as indicated here by fixations, was influenced by the emotionality of the stimulus and directed to motivationally salient cues compared to less important ones and was not specifically directed toward emotionally incongruent cues. Threatening cues, especially angry signals from faces, bodies, or scenes were looked at longer than happy cues. Similarly, participants' pupils dilated in response to different categories of social affective stimuli (faces, bodies, scenes), and were considerably larger following angry cues than happy or neutral cues. Thus, threatening cues attracted attention and induced arousal. In contrast, participants' corrugator muscle reflected the valence as shown in the facial expression of the observed, but not that of the paired body expression. However, when participants viewed a scene with a foreground body posture, both the corrugator and the zygomaticus responded exclusively to the scenes that included body expressions from multiple people, where facial expressions were blurred. We will now discuss these results in more detail starting with a discussion on participants' fixations, followed by EMG responses and pupil-size.

### Fixation duration

In Experiment 1 where participants observed congruent and incongruent face-body pairs, we showed that participants not only looked at face expressions but sampled cues from the whole body. Participants always scanned the face *and* the body. This may reflect a strategy deployed by the observers wanting to check the emotion observed in the face. In the course of development, humans learn that in social situations, and in stressful situations in particular, people try to control their face expression and put on a smile when not feeling happy or at ease (de Gelder et al., 2010). Consequently, their body language may actually be more informative. This implicit knowledge may have directed participants' attention to the body. This hypothesis is in line with our finding that bodies were longer looked at when they were emotionally incongruent vs. congruent with a happy facial expression (i.e., when the bodies were most salient). However, results from our previous EEG study showing rapid integration effects of face and body (Meeren et al., 2005) and of face and context (Righart and de Gelder, 2008a) speak against this explanation. It has been suggested previously that observers automatically attend to the body to grasp the action of the observed and prepare their own response (Kret et al., 2011a,b). The angry body gesture has most direct fight/flight consequences for the observer which is possibly why it attracted most fixations. Consequently, in Experiment 2, these action demands are most prominent in angry body gestures shown in a neutral context where the threatening foreground clearly pops out from the non-salient background scene. We believe that the fixations on the body were automatic vs. strategic and are thus better explained by the motivated attention theory.

Also our second main finding is in line with previous investigations. Participants attended mostly to threatening cues. For example, similar results were reported by Green et al. (2003), who found longer fixations on threat-related expressions, including anger, compared to threat-irrelevant expressions (such as happiness). Also, visual search studies have found that angry faces are typically detected more quickly and accurately than happy faces (Fox et al., 1987; Öhman et al., 2001; Lundqvist and Ohman, 2005). Thus, attention allocation during social interactions may reflect the need to prepare an adaptive response to social threat. Only the happy expression would signal safety and would therefore be least relevant, as indicated by shorter fixations.

### Facial EMG responses

Previous EMG studies have consistently demonstrated that individuals tend to react with congruent facial muscle activity when looking at emotional faces (Hess and Fischer, 2013). Indeed, in Experiment 1, participants' corrugator was more active when observing angry and fearful vs. happy faces, but irrespective of the body expression with which they were combined. Moreover, the zygomaticus in this experiment did not differentiate between the facial expressions. Participants always smiled to some extent, in response to all stimuli. But here facial and bodily expressions were paired, and it may be that for the EMG response (and for pupillometry), the presence of a facial expression overruled the reaction to the bodily expression. It has been questioned whether the zygomaticus and corrugator respond exclusively to face expressions or respond more broadly. Previous studies suggest the latter. For example, two earlier studies showed face expressions of emotion that were similar to the emotion expressed by either the body or the voice (Magnee et al., 2007; Tamietto et al., 2009). In a recent study participants' faces were videotaped while they observed pictures of ambiguous face expressions within a winning or losing sport context. When new participants rated the earlier participants' face expressions on valence, it turned out that the winning or losing context pulled participants rating to the positive or negative side (Aviezer et al., 2012).

Experiment 2 demonstrates that the face expression of the participant reflects the emotion from the social scenes in which all face expressions were blurred, but body expressions of the people in the background were visible. So the corrugator and zygomaticus respond to other cues than just faces. In Experiment 1, the corrugator responded to the facial- but not the body expression. It seems that for the EMG response, the presence of a face expression, even when smaller in size than a full body posture, overrules the effect of a body expression. The same might be true for the scenes: the presence of a crowd experiencing a certain emotion overrules emotional synchronization with a single emotional body posture in the front.

### Pupil dilation

Emotional arousal is a key element in modulating the pupil's response (Gilzenrat et al., 2010). In Experiments 1 and 2, we showed that participants' pupil-size was largest in response to angry faces, bodies, and scenes. Although the intensity of the emotions displayed in the happy and angry scenes was rated equally, angry scenes evoked more arousal. The happy scenes were clearly recognized as happy scenes and the angry scenes as angry scenes. These data disconfirm earlier hypotheses that pupil diameter increases when people process emotionally engaging stimuli, independent of hedonic valence (Bradley et al., 2008). Pupil dilation under constant light conditions is evoked by norepinephrine, elicited in the locus coeruleus. Different physiological manipulations (for example anxiety, noxious/painful stimulation) all increase activity in this area and result in heightened arousal and changes in autonomic function consistent with sympathetic activation (Gilzenrat et al., 2010). Our results are in line with these latter findings. Indeed, our pupils dilate in response to all emotional cues, but an enhanced effect was observed specifically following angry cues that elicit immediate arousal.

Common sense tends to hold that we read face expressions like we read single words on a page, directly and unambiguously accessing word meaning outside the sentence context. But this is not the case since a face expression is experienced differently, depending on the body expression. Body expressions are not free from contextual influences either and participants scan the body differently, depending on the face expression and on the social scene. Overall, we found that participants attended most to angry and fearful cues and their pupil-sizes increased significantly. Participants' face expressions matched the valence of the stimuli. However, when face expressions were combined with a body expression, the observed faces more strongly influenced EMG responses than the body expressions. Finally, we observed that body expressions are recognized differently depending on the social scene in which they were presented. Overall, our results show that observers' reactions to face expressions are influenced by whole body expressions and that the latter are experienced against the backdrop of the surrounding social scene. Measures hitherto assumed to be specific for viewing isolated face expressions are sensitive to the natural viewing conditions of the face. We show that when confronted with threat, be it from the face, the body, or the scene, participants' pupils dilated, their corrugator muscle became more active and they directed their gaze to the threat.

## Author contribution

Mariska E. Kret and Jeroen J. Stekelenburg were involved in data collection and filtering, Mariska E. Kret analyzed the data and prepared figures. Mariska E. Kret, Jeroen J. Stekelenburg, Karin Roelofs, and Beatrice de Gelder contributed in writing the main manuscript text.

### Conflict of interest statement

The authors declare that the research was conducted in the absence of any commercial or financial relationships that could be construed as a potential conflict of interest.

## Footnotes

^1^We also extracted fixations from ROIs on the eyes, mouth, and hands but due to the small size, observed a floor effect (*M* = 0.03, *SE* = 0.004) so these were not further analyzed.
