# Supplementary material for: Emotional signals from faces, bodies and scenes influence observers' face expressions, fixations and pupil-size
Source: Front Hum Neurosci. 2013 Dec 18;7:810. doi: 10.3389/fnhum.2013.00810 (PMC3866922; doi:10.3389/fnhum.2013.00810)
Supplement: Supplementary file 1 [file DataSheet1.PDF]

Supplementary Table

Study 1.

		Fixations on the Face		Fixations on the Body		Zygomaticus		Corrugator		Pupil size	
**Body**	**Face**	**Mean**	**Std. Error**	**Mean**	**Std. Error**	**Mean**	**Std. Error**	**Mean**	**Std. Error**	**Mean**	**Std. Error**
**Anger**	**Anger**	.624	.030	.278	.028	106.520	2.477	99.415	.955	157.858	20.989
	**Happy**	.565	.025	.309	.027	106.336	2.402	98.672	1.223	120.139	21.306
	**Fear**	.575	.028	.302	.027	111.678	4.836	98.916	1.285	125.830	24.063
**Happy**	**Anger**	.559	.029	.264	.028	109.806	3.147	100.933	1.606	114.578	24.120
	**Happy**	.569	.028	.249	.027	109.630	2.653	97.770	.877	96.436	17.874
	**Fear**	.578	.028	.241	.027	110.976	7.288	101.136	1.228	145.530	31.499
**Fear**	**Anger**	.605	.030	.265	.028	110.472	6.674	101.468	1.462	103.837	25.723
	**Happy**	.571	.030	.290	.029	113.608	6.371	96.173	1.494	111.503	23.505
	**Fear**	.594	.026	.266	.026	109.965	5.457	100.355	1.252	118.478	23.767

Study 2.

		Fixations on the Body		Fixations on the Hands		Zygomaticus		Corrugator		Pupil size	
**Body**	**Scene**	**Mean**	**Std. Error**	**Mean**	**Std. Error**	**Mean**	**Std. Error**	**Mean**	**Std. Error**	**Mean**	**Std. Error**
**Happy**	**Fear**	.408	.027	.062	.005	101.141	2.120	105.712	1.806	202.690	30.690
	**Happy**	.406	.025	.058	.006	109.044	3.409	100.612	.839	188.820	25.542
	**Neutral**	.429	.026	.056	.005	103.987	2.407	102.515	.959	130.741	19.053
**Fear**	**Fear**	.410	.024	.062	.005	101.373	2.826	107.555	2.446	168.763	30.837
	**Happy**	.399	.027	.064	.005	110.964	3.430	101.321	.856	143.326	21.225
	**Neutral**	.379	.026	.051	.005	110.838	4.356	101.854	1.023	88.579	25.541

Supplementary Table. Correlations between fixation duration and pupil-size

**Fixations on the head**									
Angry body, angry face	-.131								
Angry body, happy face	.024	.029							
Angry body, fearful face	.059	.069	.145						
Happy body, angry face	.060	.042	-.002	.000					
Happy body, happy face	-.116	-.023	.067	.031	.096				
Happy body, fearful face	.016	-.101	.132	.138	.163	.139			
Fearful body, angry face	.010	.111	.155	.153	.223	.033	.004		
Fearful body, happy face	-.240	-.159	.081	-.029	-.019	-.016	-.153	-0.452	
Fearful body, fearful face	-.015	-.018	.080	.049	.060	-.108	-.081	-.262	-.030
**Fixations on the body**									
Angry body, angry face	.079								
Angry body, happy face	.028	-.065							
Angry body, fearful face	-.018	-.064	-.041						
Happy body, angry face	-.008	-.105	-.017	-.187					
Happy body, happy face	.088	-.011	-.037	-.179	.055				
Happy body, fearful face	-.057	-.026	-.056	-.235	-.017	.029			
Fearful body, angry face	.058	-.064	-.035	-.091	.035	.159	.103		
Fearful body, happy face	.172	.132	.058	-.095	.116	.026	.141	.301	
Fearful body, fearful face	.091	.030	-.006	-.106	.129	.033	.171	.306	.042

The correlation between the length of fixations on a happy face above a fearful body and pupil size is significant at the 0.01 level (2-tailed). The more participants
